# Association Between Usage of an App to Redeem Prescribed Food Benefits and Redemption Behaviors Among the Special Supplemental Nutrition Program for Women, Infants, and Children Participants: Cross-Sectional Study

**DOI:** 10.2196/20720

**Published:** 2020-10-14

**Authors:** Qi Zhang, Junzhou Zhang, Kayoung Park, Chuanyi Tang

**Affiliations:** 1 School of Community and Environmental Health Old Dominion University Norfolk, VA United States; 2 Department of Marketing Feliciano School of Business Montclair State University Montclair, NJ United States; 3 Department of Mathematics and Statistics Old Dominion University Norfolk, VA United States; 4 Department of Marketing Old Dominion University Norfolk, VA United States

**Keywords:** mobile phone app, WIC, EBT, benefit redemption, mobile phone

## Abstract

**Background:**

The Special Supplemental Nutrition Program for Women, Infants, and Children (WIC) is one of the most important food assistance programs in the United States, serving 6.4 million low-income, eligible women, infants, and children under 5 years of age in 2019. In the program, participants are prescribed a list of food benefits, which can be redeemed in WIC-authorized stores. However, there are multiple behavioral barriers in the program and the stores that prevent participants from redeeming the benefits fully.

**Objective:**

This study aims to examine the relationship between the use of a widely used mobile phone app, WICShopper, and the redemption of the prescribed food packages.

**Methods:**

WIC administrative data were obtained from West Virginia for the period January 2019 to January 2020 and included 30,440 WIC households that had received food benefits in that period. The redemption rates of 18 WIC food benefits were compared between app users and nonapp users, that is, those who never used the app in the study period. The use behaviors were defined for the app users, including the number of active use benefit cycles, active benefit cycle rates, number of active use days in the cycle, and proportion rates of daytime use. Panel linear regressions were applied to examine how the redemption rates were related to these behaviors over time.

**Results:**

App users consistently had higher average redemption rates than nonapp users; the difference ranged from 3.6% (4.8% relative) for infant formula to 14.3% (40.7% relative) for fish. After controlling for sociodemographics, the coefficients of app use were significantly positive for all benefit categories except for WIC-eligible nutritionals. More active cycles and active days in the cycle were significantly related to redemption rates for all categories, except for frozen juice (coefficient=−0.002, *P*=.09). Daytime app access was positively associated with redemption rates for most food benefits except only a few, such as infant formula (coefficient=−0.03, *P*<.001).

**Conclusions:**

Use of the WIC app was significantly related to higher redemption rates across food benefits, although the association varied across benefit categories. More active days were positively related to benefit redemptions across food categories, and the app’s daytime use was positively associated with the redemption of most benefit categories. These findings suggest that the WIC app can be an important tool for the promotion of benefit redemption among WIC participants.

## Introduction

The Special Supplemental Nutrition Program for Women, Infants, and Children (WIC) is one of the most successful food assistance programs in the United States, serving approximately 6.4 million low-income pregnant, breastfeeding, postpartum women, infants, and children under 5 years of age in the fiscal year 2019 [1]. The WIC program plays an important role in improving the health and developmental outcomes of infants and children during critical developmental stages, promoting healthier eating, better birth outcomes, lower risk of childhood obesity, and improved household food security [2-7].

To achieve its goal, WIC certifies its enrollees for a certain number of months, that is, the certification period. During that period, an enrollee is prescribed a maximum quantity of supplemental foods, such as breakfast cereal and milk, for each monthly benefit issuance cycle, either a calendar month (eg, July 1-31) or a rolling month (eg, July 15-August 14) [8]. The prescribed food benefits can be redeemed in WIC-authorized stores before the benefits expire [9]. A WIC enrollee becomes a participant only after he or she redeems some or all of the prescribed food packages [8].

Although the WIC food benefits are free for participants, there are barriers in the retail and clinic environment that prevent participants from redeeming their benefits [9-11]. For example, it is challenging for participants to remember the specific eligible brand names or product types in each food category and identify them correctly in stores (eg, Great Value 100% apple juice is authorized in West Virginia, but Minute Maid 100% natural flavor fruit punch is not). Incorrect identification of eligible food may result in denial of the redemption in the checkout process, which may create embarrassment or frustration among WIC participants [12]. Other barriers include difficulty in remembering the benefit expiration date or clinic appointment times [13]. These barriers could contribute to the under-redemption of food benefits. As a result, only 12.6% of the WIC households in Kentucky, Michigan, and Nevada redeemed all their benefits in 2012 [14]. It is expected that when participants are not able to fully redeem the benefits they need and/or when they have negative redemption experiences, some of the unsatisfied participants may drop out of the WIC program. The national rate of participation in the WIC program steadily decreased from a peak in 2011 (64%) to the bottom in 2017 (51%) [15]. Various interventions have been implemented at the national and state levels to improve participation rate and participant satisfaction with the WIC program [16].

Electronic benefits transfer (EBT) is one of the most significant changes implemented to improve WIC administration [17]. Compared with the traditional WIC paper voucher, the EBT card, similar to a debit card, is thought to be easier and more convenient for WIC participants, vendors, and the WIC program. The implementation of the EBT system has been completed in most states; the deadline for all states to make the transition is October 1, 2020. Transition to EBT has been associated with a higher redemption of food benefits, reduced stigma at checkout, and improved overall retail experience [12,18]. More importantly, EBT allows integrating the electronic transaction system and other WIC administrative systems, which can provide an eWIC platform that makes it possible for redemption data mining and future technology innovations.

On the basis of the eWIC system, WIC apps have become a prevalent innovation across WIC state agencies. Although a federal agency funds the WIC, the program is operated at the state level in 50 states, among 34 Indian tribal organizations, in the District of Columbia, and in five US territories [19]. A recent review indicated that 17 different apps were used by participants in 37 state WIC agencies [20]. These apps include some or all of 4 main features: facilitating benefit redemption in stores, such as identifying eligible food, facilitating clinical experience, such as scheduling reminders, supplemental information (eg, recipes), and nutrition education required by the WIC program [20]. Although many WIC participants have used these apps, little research has been conducted to rigorously evaluate them, except for 1 small-scale qualitative study that demonstrated a high priority among WIC participants to access information through a WIC app [21].

Evaluating the effectiveness of WIC apps is critical for WIC agencies, participants, and researchers. First, evaluation can assist the decision making of the state agencies that have not adopted the app. Second, evaluation can help app creators identify gaps and improve app functions for WIC participants. Third, the WIC app has the potential to become a health promotion platform. Specifically, evaluation helps researchers understand WIC participants’ health-related behaviors. This information can be used to develop future intervention and health promotion programs. Finally, all these improvements can eventually help WIC participants navigate within the program more easily so that they can benefit more from it.

This study aims to evaluate the effectiveness of WIC apps to fill this knowledge gap. To the best of our knowledge, this is the first state-level WIC app evaluation in the United States that compares the redemption outcomes between WIC app users and nonapp users. We selected the WICShopper app as the research context because it has been adopted by 32 state WIC agencies, 3 Indian tribes, and 1 US territory [22], having the largest market share and the highest number of installations among all WIC apps reviewed by Weber et al [20]. In contrast, most of the other WIC apps, such as the California WIC App, are limited to 1 state and have fewer users. The WICShopper app allows the participants to check real-time benefits, eligibility of food products, WIC vendor and office locations, and recipes. The app was rated 4.5 in the Apple app store and 4.5 on Google Play, one of the highest ratings among the WIC apps, with the ratings ranging between 2 and 5; for example, Alabama WIC was rated 2.3 in the Apple app store and Arizona WIC was rated 4.7 on Google Play. Therefore, the WICShopper app is an appropriate candidate for a large-scale evaluation.

As the main purpose of the WICShopper app is to facilitate benefit redemption via multiple features, such as checking benefit balance and scanning the bar code to check eligibility, this study aims to examine how the WICShopper app usage is associated with redemption in various food benefit categories by comparing the redemption outcomes between WICShopper app users and nonapp users.

## Methods

### Study Design and Sample Selection

To examine the relationship between app usage and WIC benefit redemption, we adopted a mix of cross-sectional and longitudinal study designs. The data source was the administrative data from the West Virginia WIC agency merged with the app usage data provided by JPMA, Inc, the developer of the WICShopper app. The West Virginia WIC agency was one of the first 3 state agencies to adopt this app (in 2013).

The WIC administrative data included participants’ sociodemographics, participation status, food benefit prescriptions, and redemption records. The redemption data set contains detailed transaction information, such as transaction dates, description of the food categories, units redeemed, and the number of units redeemed. The app activity data have a timestamp that records when a user turns on the app. The WIC administrative data were linked with the app data by WIC household identification number. As 1 household normally has 1 EBT card, which can have multiple packages for multiple participants, such as a mother and her children, we used the household as the unit of analysis. The study period was from January 19, 2019, to January 18, 2020, because of the archiving cycles of the app data.

### Statistical Analyses

#### Measures

The main outcome was the redemption rate of a household in a benefit cycle, which is defined as the sum of the redeemed amount for a food category divided by the prescribed amount for that food category per household in a benefit cycle. The benefit cycle is usually a month, but it could be shorter than a month if a participant has a late visit to the WIC clinic for benefit renewal. The redemption rate was calculated using the following 18 food categories: infant meats, frozen juice, legumes, whole-grain bread, infant cereal, fish, adult cereal, WIC-eligible nutritionals, yogurt, shelf-stable juice, low-fat milk, infant fruit and vegetable, eggs, cheese, whole milk, exempt infant formula, infant formula, and cash value benefits. Each participant’s prescribed food package may vary based on participation circumstances. For example, the prescribed benefits for pregnant women are different from those prescribed for breastfeeding women [23].

The primary explanatory variable was the indicator of app users versus nonapp users, which was defined by whether a participating household had ever used the app during the entire study period or not. For example, if a WIC household used the app in March but not in April, it was still classified as an app user in the year. Secondary explanatory variables calculated among the app users were: the *number of active app cycles*, defined as the number of benefit cycles in which the participant actively used the app; *active cycle rate*, defined as the percentage of active app cycles out of all benefit cycles for that household in the study period; the *number of active app days*, defined as the number of days in a benefit cycle the participant used the app; and *daytime rate*, defined as the percentage of app usage from 6 AM to 5 PM in a benefit cycle. As each household has a unique ID to log into the app, activity was recorded at the household level, regardless of how many individual users accessed it.

The control variables included the participants’ demographic information: race (White or non-White), whether the household had infant, child, or female participants, and the number of WIC participants and overall household size, including members not participating in WIC. According to the US Census, 92% of residents in West Virginia in 2019 were non-Hispanic Whites [24]. In our study sample, 26,630 of the 30,440 households (87.5%) were non-Hispanic Whites. If the non-White households were further broken down, the sample size would be too small to be representative of the specific groups. Therefore, we combined all non-Whites into 1 racial or ethnic group. These control variables were at the household level; in the case of a household with multiple participants, for example, a mixed-race household, we used the race of the eldest WIC participant.

#### Statistical Methods

We first estimated the descriptive statistics for sociodemographics and app usage. Differences in sociodemographics between app users and nonapp users were then tested using Chi-square or Wilcoxon rank-sum tests. App usage activities were also tested across the sociodemographics. Next, we estimated the average redemption rates across food categories and tested the difference in the redemption rates between app and nonapp users by using the Wilcoxon rank-sum test. The absolute difference in the redemption rates between app and nonapp users was calculated as the app users’ average redemption rate minus that of nonapp users, whereas the relative difference was calculated as the absolute difference divided by the average nonapp users’ redemption rate. We divided the 18 food groups into low, medium, and high redemption groups with 6 food categories per group. We plotted the average redemption rates by app usage and groups with absolute and relative differences.

To examine the relationship between the redemption rate and app usage behaviors, we applied 3 sets of linear regressions across food categories. First, we regressed the average annual redemption rate on the app usage indicator among all households (app users and nonusers) in the study period. We then focused only on app users with additional analyses. The second set of regressions examined how the average annual redemption rate was related to the number of active app cycles or the active app cycle rates. The third set of regressions were panel analyses across benefit cycles. Random-effects models with an unbalanced panel were employed in our empirical evaluation of WIC participants’ redemption behaviors. Sociodemographics were controlled in all sets of regressions. Statistical significance was set at *P*<.05, and the R software was employed to conduct the statistical analyses [25]. This study was approved by the institutional review board of the Old Dominion University.

## Results

### Descriptive Statistics

Table 1 compares the descriptive statistics of the WIC participants’ sociodemographics between app users and nonapp users. The entire study sample included 30,440 WIC households in West Virginia during the study period. Non-Hispanic White accounted for 87.48% (26,630/30,440) of the participating households. The prevalence rates of infants, children, and women were 29.05% (8844/30,440), 76.17% (23,187/30,440), and 70.85% (21,567/30,440), respectively. Of the 30,440 households, 13,925 (45.75%) had 2 WIC participants; households with 1 WIC participant and 3 or more participants accounted for a similar percentage of the study sample (8015/30,440, 26.33%, and 8500/30,440, 27.92%, respectively). On average, the household size was 3.84. Of the study population, 72.26% (21,996/30,440) of the households used the WICShopper app at least once in the study period. Non-Hispanic White households had a significantly higher percentage of app users (19,496/26,630, 73.21%) than non-White households (2500/3810, 65.62%; *P*<.001). The percentage of app users increased when there were more participants in a household (5070/8015, 63.26%; 10,358/13,925, 74.38%; and 6568/8500, 77.27% for households with 1, 2, and 3 or more participants, respectively; *P*<.001), which is consistent with the result that app users, on average, tended to have a larger household (*P*<.001).

**Table 1:** Descriptive statistics of the sociodemographics from participants of the Special Supplemental Nutrition Program for Women, Infants, and Children in West Virginia (N=30,440).

Variables		Whole sample, n	Nonapp users (n=8444), n (%)	App users (n=21,996), n (%)		*P* value^a^
**Racial group**					<.001	
	White	26,630	7134 (26.79)	19,496 (73.21)		
	Non-White	3810	1310 (34.38)	2500 (65.62)		
**Did the household have an infant participant?**					<.001	
	Yes	8844	2092 (23.65)	6752 (76.35)		
	No	21,596	6352 (29.41)	15,244 (70.59)		
**Did the household have a child participant?**					.92	
	Yes	23,187	6436 (27.76)	16,751 (72.24)		
	No	7253	2008 (27.69)	5245 (72.31)		
**Did the household have a female participant?**					<.001	
	Yes	21,567	5266 (24.42)	16,301 (75.58)		
	No	8873	3178 (35.82)	5695 (64.18)		
**Number of WIC^b^ participants**					<.001	
	1	8015	2945 (36.74)	5070 (63.26)		
	2	13,925	3567 (25.62)	10,358 (74.38)		
	≥3	8500	1932 (22.73)	6568 (77.27)		
Household size (people), mean (SD)		3.84 (1.60)	3.73 (1.69)	3.88 (1.56)		<.001

^a^Statistical tests performed: Chi-square test of independence and Wilcoxon rank-sum test.

^b^WIC: The Special Supplemental Nutrition Program for Women, Infants, and Children.

### App Usage Behaviors

Table 2 presents the pattern of WIC app users’ app usage behavior across key demographic variables. At the aggregated level, these app users actively used the WIC app in 7.93 benefit cycles, which was equivalent to 85.8% of all their benefit cycles in the study period. App users activated the app for 4.05 days per monthly benefit issuance cycle, which is, on average, equivalent to once per week. On average, 63.3% of app usage behaviors occurred during the daytime. White households were more active in app usage than non-White households, except for the number of active app days (*P*=.70).

In addition, households with infant or woman participants tended to be more active in terms of using the WIC app in a prescribed benefit cycle. For example, households with infants and households with woman participants used the WIC app about 4.38 days and 4.11 days in 1 benefit cycle, respectively, whereas households without infants and households without woman participants averaged 3.91 days and 3.90 days, respectively (*P*<.001). In addition, households with children were less active in using the WIC app, averaging 4.01 days of WIC app use in a prescribed benefit cycle compared with 4.19 days for households without children (*P*<.001).

**Table 2:** The Special Supplemental Nutrition Program for Women, Infants, and Children app users’ characteristics at the household level (n=21,996). Statistics are presented as mean (SD), and the tests performed were the Wilcoxon rank-sum test and Kruskal-Wallis test.

Variables		Number of active app cycles		*P* value		Active cycle rate, n (%)		*P* value		Number of active app days		*P* value		Daytime rate, n (%)		*P* value
Whole sample		7.93 (3.35)		N/A^a^		85.8 (23.5)		N/A		4.05 (2.05)		N/A		63.3 (22.8)		N/A
**Race group**			.005		N/A		.02		N/A		.70		N/A		<.001	
	White	7.97 (3.34)				85.9 (23.4)				4.05 (2.04)				63.6 (22.8)		
	Non-White	7.78 (3.38)				84.6 (24.2)				4.08 (2.09)				61.1 (22.9)		
**Did the household have an infant participant?**			<.001		N/A		.40		N/A		<.001		N/A		<.001	
	Yes	7.94 (3.07)				85.7 (23.5)				4.38 (2.08)				62.4 (22.2)		
	No	7.95 (3.46)				85.8 (23.5)				3.91 (2.01)				63.7 (23.1)		
**Did the household have a child participant?**			<.001		N/A		.007		N/A		<.001		N/A		<.001	
	Yes	8.40 (3.23)				86.1 (23.2)				4.01 (2.03)				64.2 (22.6)		
	No	6.50 (3.29)				84.6 (24.2)				4.19 (2.07)				60.3 (23.3)		
**Did the household have a woman participant?**			.20		N/A		.90		N/A		<.001		N/A		<.001	
	Yes	7.98 (3.31)				86.0 (23.2)				4.11 (2.04)				62.9 (22.5)		
	No	7.87 (3.44)				85.2 (24.4)				3.90 (2.05)				64.5 (23.6)		
**Number of WIC^b^** **participants**			<.001		N/A		<.001		N/A		<.001		N/A		<.001	
	1	6.65 (3.63)				84.4 (24.8)				3.82 (2.07)				62.3 (24.4)		
	2	8.13 (3.20)				85.3 (23.6)				3.94 (1.95)				62.5 (22.7)		
	≥3	8.66 (3.04)				87.5 (22.1)				4.42 (2.13)				65.3 (21.7)		

^a^N/A: not applicable.

^b^WIC: The Special Supplemental Nutrition Program for Women, Infants, and Children.

### Redemption Rates and App Usages

Multimedia Appendices 1-3 present the average redemption rates of 18 WIC food categories among app users and nonapp users. We divided the food benefits into 3 groups (low, medium, and high redemption) according to the rankings of their redemption rates from the lowest to highest (infant meat, 27.4%, to infant formula, 77.9%). In all groups, app users had a higher redemption rate than nonusers, and the difference was statistically significant in all food categories except in WIC-eligible nutritionals (*P*=.12).

Multimedia Appendices 4 and 5 compare the differences in the redemption rates between app users and nonapp users across food benefits. First, the 18 food benefits were divided into 3 groups based on the redemption rates (6 benefits in each group: low, medium, and high redemption rate groups). The figure marked these food benefits with red, yellow, and green bars, respectively. These bars were then ordered based on the magnitude of the differences, from the smallest to the largest difference. In Multimedia Appendix 4, the 3 groups of colored bars were almost evenly distributed. However, in Multimedia Appendix 5, high (green bars) and medium (yellow bars) redemption groups were more concentrated toward the left end of the axis, whereas the low redemption groups (red bars) were more concentrated toward the right end of the axis. For example, the bottom 5 food groups in terms of relative differences were either high or medium redemption groups (green or yellow bars toward the left: infant formula, exempt infant formula, WIC-eligible nutritionals, low-fat milk, and whole-grain bread), whereas the top 5 groups in relative difference were all in low redemption groups (red bars toward the right: fish, frozen juice, infant cereal, infant meats, and legumes). The patterns indicate that the WIC app may be more helpful in improving redemption rates relatively for the food benefits with lower redemption rates, that is, less-popular food benefits.

The regression results for all food categories are presented in Tables 3 and 4. Model 1 examined the association between the annual redemption rate and the app usage indicator for all households, including app users and nonapp users. Models 2 and 3 focused only on app users, with the goal of examining the effect of the number of active app cycles and the percentage of active app cycles in the study period. Models 4 and 5 in Table 4 are panel analyses of the relationship between redemption rates and the number of app active days, and the percentage of app usage in the daytime across benefit cycles. All models controlled sociodemographic variables.

**Table 3:** Linear regression of the Special Supplemental Nutrition Program for Women, Infants, and Children benefit redemption rates on app activities by food categories in West Virginia participating households (N=30,440 and n=21,996 for app users only).

Variables (food category)	Whole sample^a^			App users only^a^					
	App use (Yes=1; No=0); model 1			Number of active app cycles; model 2			Active cycles rate; model 3		
	Coefficient	SE	*P* value	Coefficient	SE	*P* value	Coefficient	SE	*P* value
Infant meats	0.06	0.03	.045	0.02	0.005	<.001	0.30	0.05	<.001
Frozen juice	0.08	0.007	<.001	−0.002	0.001	.09	0.24	0.01	<.001
Legumes	0.08	0.005	<.001	0.007	0.001	<.001	0.29	0.01	<.001
Whole-grain bread	0.07	0.005	<.001	0.006	0.001	<.001	0.27	0.01	<.001
Infant cereal	0.11	0.008	<.001	0.02	0.001	<.001	0.25	0.02	<.001
Fish	0.14	0.02	<.001	0.007	0.003	.02	0.47	0.04	<.001
Adult cereal	0.07	0.004	<.001	0.005	0.001	<.001	0.29	0.009	<.001
WIC^b^-eligible nutritionals	0.04	0.03	.17	0.01	0.005	.046	0.23	0.06	<.001
Yogurt	0.10	0.005	<.001	0.004	0.001	<.001	0.32	0.01	<.001
Shelf-stable juice	0.08	0.005	<.001	0.01	0.001	<.001	0.30	0.01	<.001
Low-fat milk	0.08	0.005	<.001	0.01	0.001	<.001	0.30	0.009	<.001
Infant fruit and vegetable	0.10	0.007	<.001	0.02	0.001	<.001	0.25	0.01	<.001
Eggs	0.10	0.004	<.001	0.01	0.001	<.001	0.33	0.008	<.001
Cheese	0.09	0.004	<.001	0.01	0.001	<.001	0.34	0.009	<.001
Cash value benefit	0.10	0.004	<.001	0.01	0.001	<.001	0.30	0.008	<.001
Whole milk	0.08	0.007	<.001	0.02	0.001	<.001	0.29	0.01	<.001
Exempt infant formula	0.04	0.01	.001	0.006	0.002	.005	0.12	0.03	<.001
Infant formula	0.03	.005	<.001	0.009	0.001	<.001	0.09	0.01	<.001

^a^Sociodemographics controlled: race, whether the household had an infant, child, female participants, the number of the Special Supplemental Nutrition Program for Women, Infants, and Children participants in a household, and the overall household size (people).

^b^WIC: The Special Supplemental Nutrition Program for Women, Infants, and Children.

**Table 4:** Panel regression of the Special Supplemental Nutrition Program for Women, Infants, and Children benefit redemption rates on app activities by food categories in West Virginia participating households (n=21,996 for app users only).

Variables (food category)	Number of active app days^a^, model 4			Daytime rate^a^, model 5		
	Coefficient	SE	*P* value	Coefficient	SE	*P* value
Infant meats	0.02	0.003	<.001	−0.006	0.02	.79
Frozen juice	0.02	0.001	<.001	0.01	0.006	.04
Legumes	0.02	0.001	<.001	0.01	0.004	.003
Whole-grain bread	0.02	<0.001	<.001	0.02	0.004	<.001
Infant cereal	0.03	0.001	<.001	0.02	0.008	.007
Fish	0.03	0.002	<.001	0.02	0.02	.31
Adult cereal	0.03	<0.001	<.001	0.008	0.004	.04
WIC^b^-eligible nutritionals	0.02	0.002	<.001	0.02	0.02	.49
Yogurt	0.02	0.001	<.001	0.01	0.005	.03
Shelf-stable juice	0.03	0.001	<.001	0.01	0.005	.004
Low-fat milk	0.03	<0.001	<.001	0.006	0.003	.10
Infant fruit and vegetable	0.03	0.001	<.001	0.02	0.007	.03
Eggs	0.03	<0.001	<.001	0.02	0.004	<.001
Cheese	0.02	<0.001	<.001	0.02	0.004	<.001
Cash value benefit	0.02	<0.001	<.001	0.02	0.003	<.001
Whole milk	0.03	0.001	<.001	−0.005	0.006	.35
Exempt infant formula	0.01	0.001	<.001	−0.02	0.01	.10
Infant formula	0.008	0.001	<.001	−0.03	0.005	<.001

^a^Sociodemographics controlled: race, whether the household had an infant, child, and female participants, the number of The Special Supplemental Nutrition Program for Women, Infants, and Children participants in a household, and the overall household size (people).

^b^WIC: The Special Supplemental Nutrition Program for Women, Infants, and Children.

In Model 1, we observed that app usage was significantly associated with higher redemption rates for almost all food categories except WIC-eligible nutritionals (coefficient=0.04, *P*=.17). After adjusting for sociodemographics, using an app generated the lowest increase in the average redemption rate, as in the case of 3 percentage points in infant formula, and the highest increase, as in the case of 14 percentage points in fish. In app users (Model 2), a higher number of active app cycles was significantly associated with a higher redemption rate, except in the category of frozen juice (48 oz; coefficient=−0.002, *P*=.09). The largest number of active app cycles was observed for infant fruits and vegetables, infant meats, infant cereals, and whole milk (coefficient=0.02, *P*<.001), whereas the smallest coefficients were observed for yogurt (coefficient=0.004, *P*<.001). These results indicated that having 1 additional active app cycle was associated with a 0.4 to 2 percentage point increase in redemption rates. Similarly, increasing the active cycle rates by 10% was associated with a 0.9 to 4.7 percentage point increase (infant formula vs fish) in redemption rates (Model 3).

In Table 4, Models 4 to 5 present how app activity per cycle was associated with the redemption rates of the food benefits. Model 4 indicates that the number of days of the WICShopper app usage was positively associated with the redemption rates, with statistical significance in all food categories (*P*<.001). Here, again, the smallest and largest coefficients were observed in infant formula and fish. Model 5 presents the coefficients of daytime usage. The percentage of use of the WIC app during the daytime was significantly associated with a higher redemption rate for most food categories, except for infant meats, fish, WIC-eligible nutritionals, low-fat milk, whole milk, and exempt infant formula. Notably, using the app during the daytime was associated with lower redemption rates in infant formula (coefficient=−0.03, *P*<.001).

Overall, the regression results suggest that the use of the WICShopper app was significantly associated with higher redemption rates. However, the positive app effect may diminish with the popularity of the various food benefits; that is, app usage may not have a large effect on the redemption of some popular food benefits (eg, infant formula).

## Discussion

### Principal Findings

To the best of our knowledge, this is the first state-level study examining the association between WIC app usage and WIC benefit redemption. We found a significant positive association between app usage and redemption rates in all food benefit categories except for WIC-eligible nutritionals. These results are encouraging for WIC policymakers who had concerns about the prevalent underredemption of benefits among WIC participants [14,26,27]. In general, app user redemption rates were at least 10% higher than those of nonusers in all food categories except for WIC-eligible nutritionals, exempt infant formula, and regular infant formula. Given that WIC-eligible nutritionals and exempt infant formula require medical documentation to be prescribed, infant formula was the only regular food benefit for which redemption was not related to app usage on a large scale, although the association was still statistically significant. Given the positive findings of this study, state WIC agencies may consider increasing access to the app for participants.

Several factors may explain the weak relationship between app usage and infant formula. First, infant formula was the most redeemed food category, with an average redemption rate of 77.9%. Given that the redemption rate was already very high or *near the ceiling*, using an app may not be able to further improve the redemption rate. Thus, this ceiling effect may diminish the scale of the app effect on this redemption. Second, infant formula is the most valuable food benefit for participants, who may treat the redemption of the formula as their highest priority. Usually, each state agency contracts with only one formula brand, such as Similac in West Virginia. On the basis of the findings from behavioral economics, the high priority of obtaining the infant formula, and the ease in identifying the formula product may reduce the need for app assistance to facilitate redemption [12,28]. As a result, app usage is unlikely to change the infant formula redemption rate on a large scale.

In addition to infant formula, app users had much higher redemption rates than nonapp users in the rest of the regular food categories. The relative difference in redemption rate between app users and nonusers was most evident in the least-redeemed food categories, including infant meats, frozen juice, legumes, infant cereal, and fish. There are several potential theoretical explanations for these results. First, the *temptation bundling* theory suggests that combining the less-preferred activities with preferred activities may increase both activities [29]. With the WICShopper app, participants can easily check their remaining benefits for all food categories in their prescribed food package as a bundle. Creating a *bundling effect* within the app may motivate participants to redeem all benefits together, even though some of the bundled food benefits may not be among their most preferred. On the other hand, nonapp users may not have the opportunity to view all the food benefit balances in real time and in a convenient way. As a result, some benefits may remain unredeemed. Second, using the app can help identify the eligible brand and product type more easily for certain food categories, such as infant cereal, which may reduce redemption barriers [26]. However, for other food categories with fewer eligible brands, such as infant formula, the app may have a limited impact on brand choices. Finally, the difference may be caused by factors other than app usage. App users and nonapp users may have different preferences and behavioral patterns extrinsic to the app itself. For example, app users may care more about their welfare than nonusers. Thus, they are more likely to download the app and redeem more benefits than nonusers, even if certain food categories are not popular choices. Although extensive research has studied the self-selection bias in WIC participation, no research has examined the potential self-selection bias in WIC app usage, which should be addressed in future studies using a randomized experiment or statistical methods, such as propensity score or instrumental variable [30,31].

The panel analyses among the app users further examined how the app activities across cycles may be associated with redemption rates. The frequency and timing of app usage per cycle (eg, in the daytime) were associated with redemption rates in most food benefits. Given the app’s multiple functions, using the app may not only improve the participants’ shopping experience but also help with their food planning, as they can check the remaining balance at any time. The ability to check the benefit balance was ranked as the most useful app feature by WIC participants [32]. About 82% of participants rated it as *very useful*, but lower percentage of participants rated other features as *very useful*, such as checking the WIC shopping guide (73%), checking eligible food products (71%), or locating WIC-authorized stores (62%) [32]. Therefore, the WIC app may help engage participants in the WIC program and help them better utilize the resources the program provides in general.

The transition to EBT is deemed one of the most significant technological changes in the WIC program over the past 4 decades [14]. One of the main purposes of EBT transition is to facilitate the benefit redemption experience. However, a recent state-level study suggests that EBT transition did not significantly change the redemption of produce or infant formula [18]. Although the EBT system itself may not exert a significant impact on every aspect of the WIC program, it provides a necessary base for further technological innovations to improve WIC operations and participant experience [20,33]. For example, we found that WIC app adoption and frequent app usage were positively associated with the redemption of all food benefits, except WIC-eligible nutritionals, in West Virginia. Researchers may develop more EBT-based or app-based interventions to improve participant experience in the WIC program [34].

### Limitations

Although this is the first state-level study to provide empirical evidence of positive relationships between app usage and WIC benefit redemptions, the results need to be interpreted carefully because of several limitations. First, the pooled analyses were essentially cross-sectional, so only the association instead of the causality can be concluded. More studies are needed to examine the determinants of app adoption among WIC participants and how self-selection bias can affect study results. More rigorous study designs, such as a field experiment or advanced statistical methods (such as propensity score or instrumental variable), may help address the self-selection bias of app users so that the real app effect can be more accurately estimated. Second, because there are a large number of users from all states adopting the WICShopper app, app developers do not archive detailed app activity data, such as the pages visited or the sequence of the clicks. Therefore, the specific features the app users used are not known. This creates challenges in linking app feature usage directly to redemption behaviors.

Moreover, there is no information about whether the participating households ever download the app or even have smartphones. Finally, WIC administrative data have a limited number of sociodemographic variables that can be controlled in regression models. As applicants can be eligible for WIC if they are participants in other means-tested social welfare programs, such as Medicaid or the Supplemental Nutrition Assistance Program (SNAP) [35], income was not required to be reported in the WIC database, which resulted in 85.3% (25,973/30,440) of the income data being missing. Therefore, we did not control for income in the analyses. As redemption behaviors can be influenced by other individuals, household, and community factors, more insights might be gained by linking our data with other state-level data sets, such as SNAP participant data or Medicaid participant data. However, such a step would require additional administrative reviews within other state agencies, which could be challenging. Despite the above limitations and challenges, this study employed the best available data set to generate important preliminary findings on the impact of technology on WIC participation—app users had greater redemption rates than nonapp users.

### Conclusions

Employing WIC administrative data and WIC app usage data from West Virginia, this study provides important evidence about the positive association between WIC app usage and the redemption of WIC food benefits. These results can help the decision-making process in state WIC agencies that have not yet adopted the app in their program operations. Moreover, because more frequent app usage is related to higher redemption rates in most food categories, state agencies may seek to promote more app downloads and usage among WIC participants. Finally, as most state agencies will transition to the EBT system by October 2020 (although some states have received waivers), more app-based innovations may be developed and implemented to improve the participant experience, through which WIC participant retention can be improved. To maximize the benefits of the WIC app, state WIC agencies, app developers, and retail stores need to work together closely to ensure that the latest approved food products and redemption data are integrated, updated, and shared in a timely and efficient manner.

## Appendix

Multimedia Appendix 1 Average annual redemption rates among all subjects, app users, and nonapp users (low redemption food group; *: *P*<.001).

Multimedia Appendix 2 Average annual redemption rates among all subjects, app users, and nonapp users (medium redemption food group; **P*<.001; WIC: The Special Supplemental Nutrition Program for Women, Infants, and Children).

Multimedia Appendix 3 Average annual redemption rates among all subjects, app users, and nonapp users (high redemption food group; **P*<.001).

Multimedia Appendix 4 Absolute differences in the redemption rates between app users and nonapp users.

Multimedia Appendix 5 Relative differences in the redemption rates between app users and nonapp users.

## Abbreviations

EBT: electronic benefits transfer
SNAP: Supplemental Nutrition Assistance Program
WIC: The Special Supplemental Nutrition Program for Women, Infants, and Children
