# Correction: Association Between Usage of an App to Redeem Prescribed Food Benefits and Redemption Behaviors Among the Special Supplemental Nutrition Program for Women, Infants, and Children Participants: Cross-Sectional Study

**DOI:** 10.2196/25073

**Published:** 2020-10-21

**Authors:** Qi Zhang, Junzhou Zhang, Kayoung Park, Chuanyi Tang

**Affiliations:** 1 School of Community and Environmental Health Old Dominion University Norfolk, VA United States; 2 Department of Marketing Feliciano School of Business Montclair State University Montclair, NJ United States; 3 Department of Mathematics and Statistics Old Dominion University Norfolk, VA United States; 4 Department of Marketing Old Dominion University Norfolk, VA United States

In “Association Between Usage of an App to Redeem Prescribed Food Benefits and Redemption Behaviors Among the Special Supplemental Nutrition Program for Women, Infants, and Children Participants: Cross-Sectional Study” (JMIR Mhealth Uhealth 2020;8(10):e20720) the authors noted one error.

In the reference list, the author of Reference 22 was incorrectly listed as “Juvenile Products Manufacturers Association”. The correct author is “JPMA, Inc”, an unrelated organization.

The correction will appear in the online version of the paper on the JMIR Publications website on October 21, 2020, together with the publication of this correction notice. Because this was made after submission to PubMed, PubMed Central, and other full-text repositories, the corrected article has also been resubmitted to those repositories.
