# Supplementary figures and images for: Association Between Usage of an App to Redeem Prescribed Food Benefits and Redemption Behaviors Among the Special Supplemental Nutrition Program for Women, Infants, and Children Participants: Cross-Sectional Study
Source: JMIR Mhealth Uhealth. 2020 Oct 14;8(10):e20720. doi: 10.2196/20720 (PMC7593867; doi:10.2196/20720)

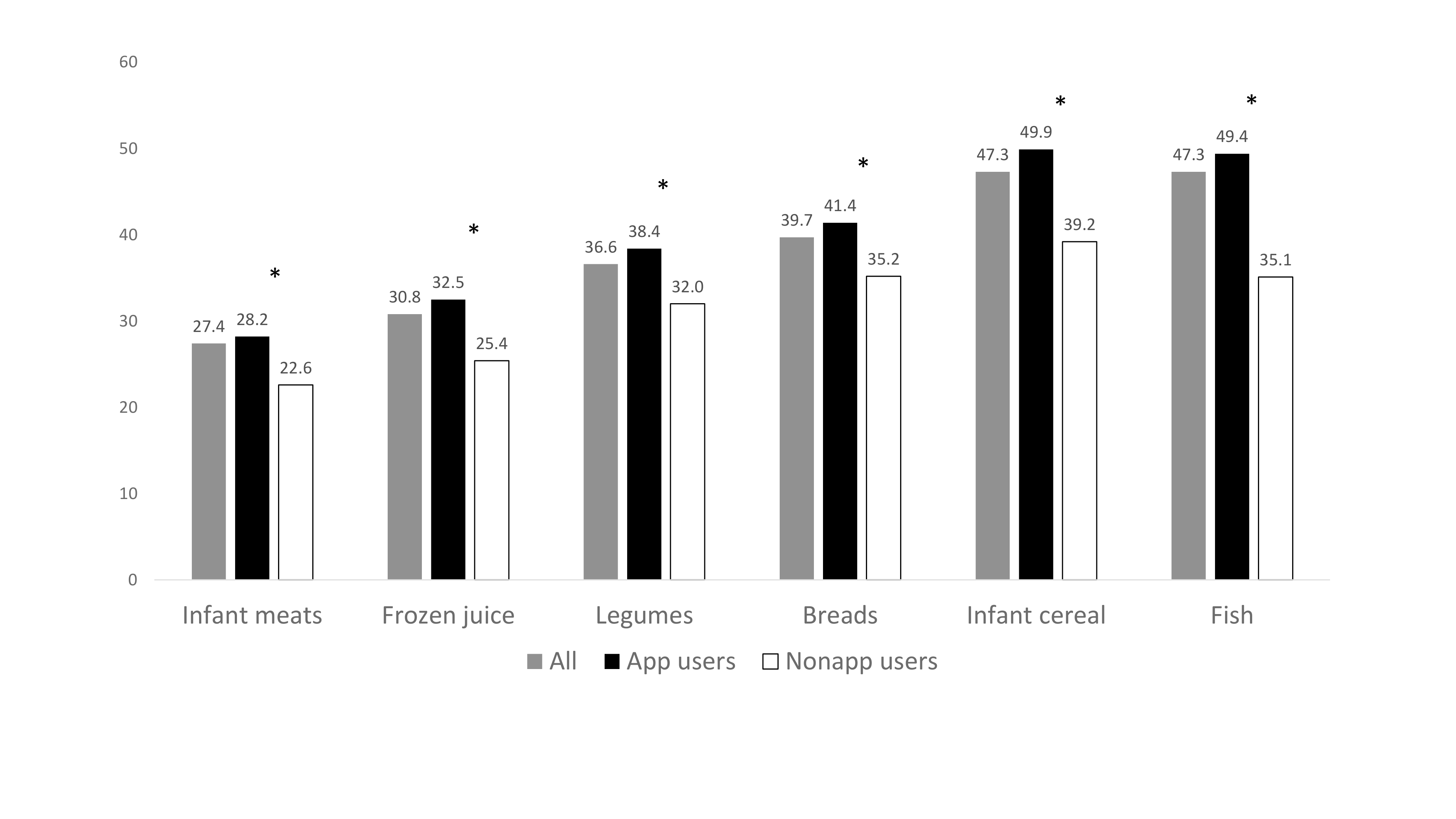

Supplement: Multimedia Appendix 1 [file mhealth_v8i10e20720_app1.png]

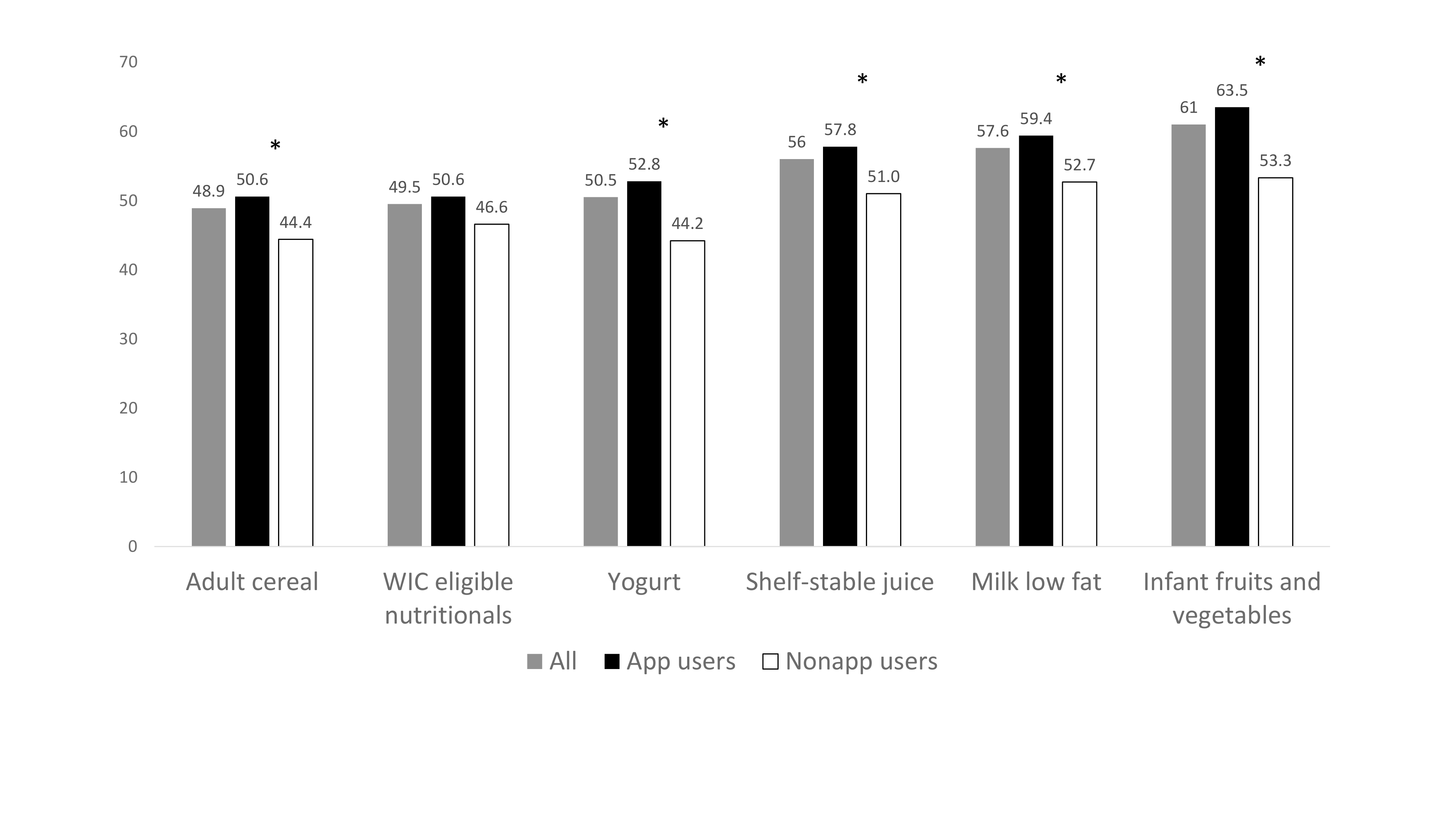

Supplement: Multimedia Appendix 2 [file mhealth_v8i10e20720_app2.png]

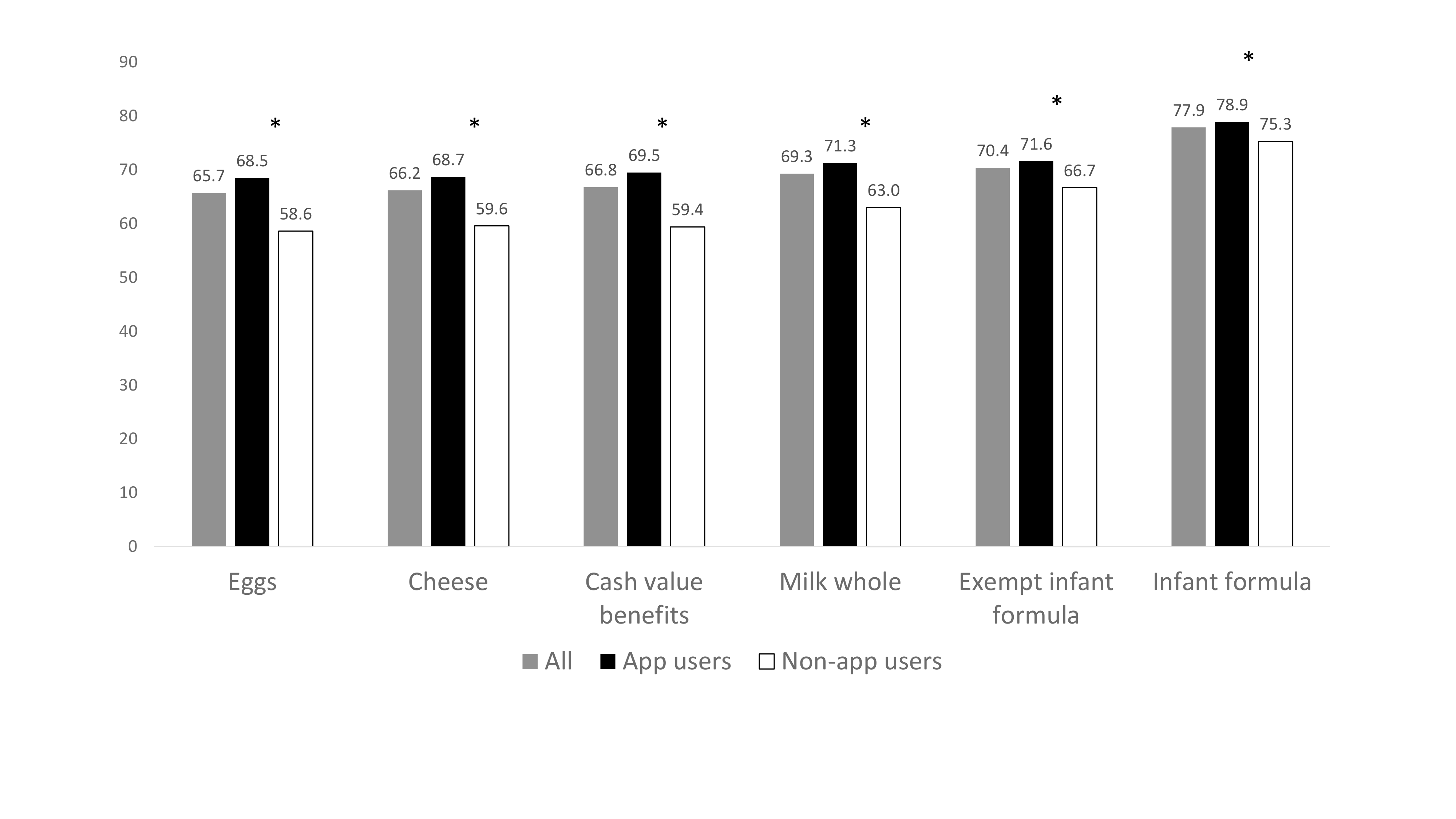

Supplement: Multimedia Appendix 3 [file mhealth_v8i10e20720_app3.png]

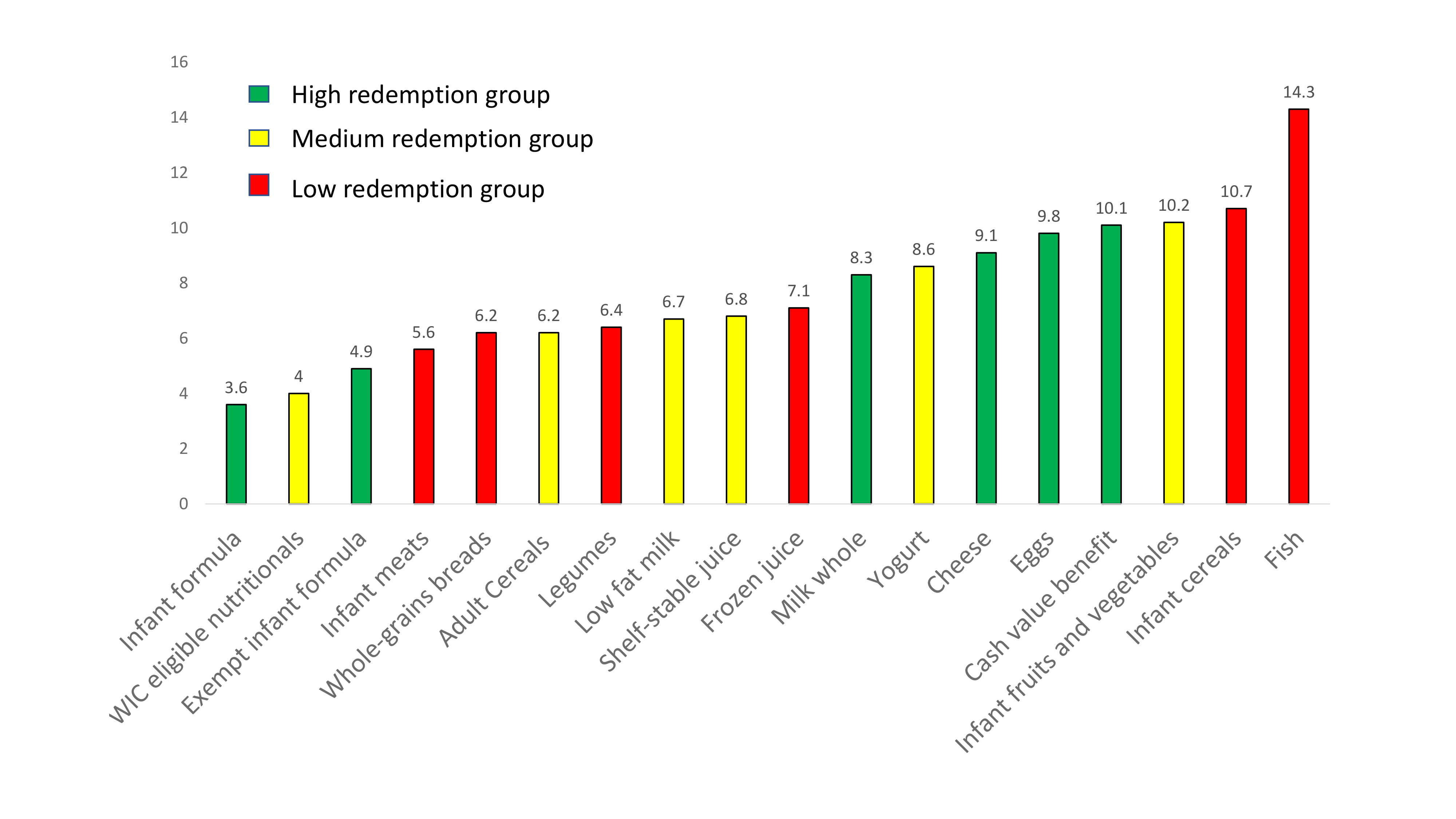

Supplement: Multimedia Appendix 4 [file mhealth_v8i10e20720_app4.png]

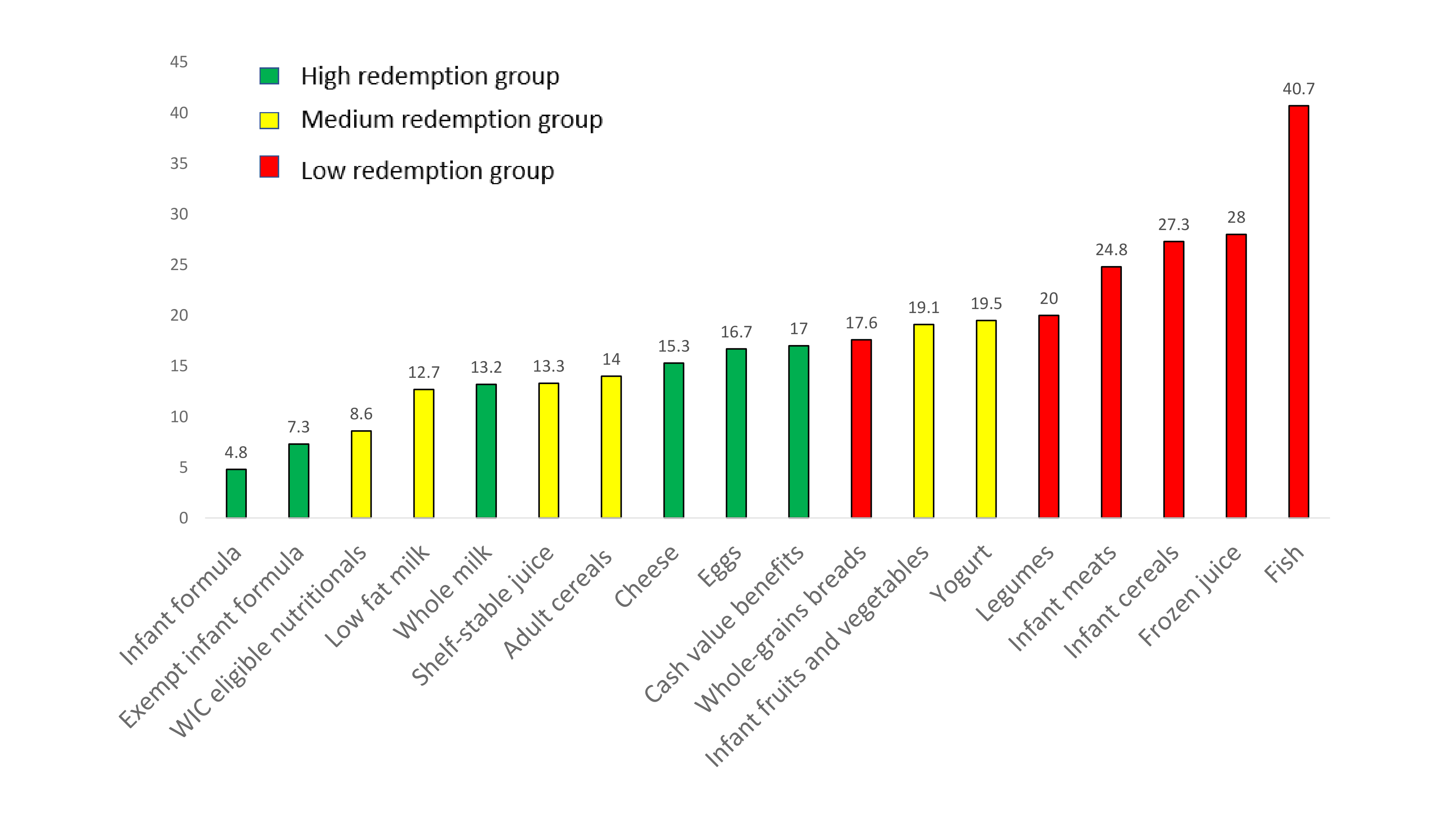

Supplement: Multimedia Appendix 5 [file mhealth_v8i10e20720_app5.png]
